# Representative Claims in Healthcare: Identifying the Variety in Patient Representation

**DOI:** 10.1007/s11673-018-9861-x

**Published:** 2018-06-07

**Authors:** Hester M. van de Bovenkamp, Hans Vollaard

**Affiliations:** 1 Erasmus School of Health, Policy & Management, Erasmus University Rotterdam, P.O. Box 1738, 3000 DR Rotterdam, The Netherlands; 2 Utrecht School of Governance, Utrecht University, Utrecht, The Netherlands

**Keywords:** Healthcare, Patient organizations, Representation, Representative claims

## Abstract

In many countries patient involvement is high on the healthcare policy agenda, which includes patient representation in collective decision-making. Patient organizations are generally considered to be important representatives of patients. Other actors also claim to represent patients in decision-making, such as politicians, healthcare professionals, and client advisory councils. In this paper we take a broad view of patient representation, examining all the actors claiming to represent patients in the Dutch debate on the decentralization of care. We conclude that variety in forms of representation could help do justice to the variety of patient preferences. In addition we conclude that in order to ensure the democratic quality of patient representation, actors making representative claims have to reflect on how their claims relate to each other and how they can ensure authorization and accountability in the representative relationship with those they claim to represent.

## Introduction

As a matter of principle, it is wrong to let municipalities decide whether a child has autism or is anorexic. Or that the municipality can say to a child with ADHD that he should exercise a bit more. Children have the right to medical care. However, children with psychiatric conditions are no longer [automatically] insured as the municipality decides on and pays for their care. (Child Psychiatrist arguing against the decentralization of mental healthcare for children, taken from Müller 2013).

In many countries, patient involvement in healthcare is high on the policy agenda (Clarke et al. 2007; Kendall et al. 2011; Dwarswaard and van de Bovenkamp 2015). Patients are increasingly expected to become active in their own care, for example by choosing their healthcare provider and actively managing their disease.

Patients are not only expected to be involved in their own care. As this symposium shows, the participation or representation of patients is also an important issue in collective healthcare decision-making. Examples of such decision-making include guideline development, setting research agendas, supervision, and government policymaking (van de Bovenkamp 2010). In this paper we focus on patient representation on this collective level. Although current literature presents us with interesting insights into representation on this level (Baggott, Allsop, and Jones 2005; Barbot 2006; Baggott and Forster 2008; Epstein 2008; Jones 2008; van de Bovenkamp, Trappenburg, and Grit 2010) the issue of *who* can represent patients remains underexplored.

It is often assumed that patients’ own organizations (POs) are the (best) ones to represent patients. However, research into POs indicates certain problems concerning their representation efforts (Baggott and Forster 2008; Akrich et al. 2013; van de Bovenkamp, Trappenburg, and Grit 2010). For instance, often only highly educated professionalized people acting on behalf of POs are representing patients in formal decision-making processes. These people may be patients themselves who participate in all kinds of decision-making processes and have thus acquired a lot of knowledge on these processes. Some POs employ professionals who are not patients themselves. The question is whether these professional patients or employees can fully represent the interests of diverse groups of patients who might suffer from other diseases or from the same diseases but in different conditions (Baggott and Forster 2008; van de Bovenkamp 2010; van de Bovenkamp and Zuiderent-Jerak 2015).

Recent political science literature focusing on representation shows that it is important to move beyond a focus on the usual suspects when studying representation. This literature argues that when studying representation, one should examine all the actors making representative claims in the public debate (Urbinati 2006; Saward 2010; Montanaro 2012; van de Bovenkamp and Vollaard 2017). Representative claims can be expressed both explicitly and implicitly, for instance actors arguing for a certain issue could state that it is in the best interest of a certain group. This means that actors for whom representation is not a core task, such as religious leaders claiming to represent their followers in certain public debates or the professional arguing for his patients in the quote we started out with, should also be considered representatives (Saward 2010). Seen this way, there are other actors besides POs claiming to represent patients in the public debate. One can think of politicians and healthcare professionals in this respect. To obtain an in-depth view of patient representation, these other actors should be included in the analysis. This is especially important because some of these actors assert that they could be better able to cater for groups of patients who are not inclined to speak up themselves than professional patients can (van de Bovenkamp and Zuiderent-Jerak 2015).

To determine the contribution different actors make to representing patients, it is important to gain insight into the democratic quality of their claim and thereby be able to say more about the legitimacy of patient representation (Baggott and Forster 2008). Therefore, we examine the responsiveness of actors making representative claims. Responsiveness focuses on the relationship between those who claim to represent and the patients they claim to represent (Urbinati and Warren 2008; van de Bovenkamp and Vollaard 2017). We explore the *authorization* mechanisms in place (the way representatives are selected or directed by those they claim to represent) and the mechanisms used by representatives to *account* for their actions (how they explain and justify their conduct to the represented) (Montanaro 2012; van de Bovenkamp and Vollaard 2017). Authorization and accountability mechanisms can be formal and informal and include elections, signing petitions, deliberation, joining protests, starting or terminating membership of an organization, expressions of trust, accounting for one’s actions in the public debate or at meetings with members, or making plans, financial accounts, or annual reports publicly available (Urbinati and Warren 2008; Montanaro 2012).

When identifying and analysing representation broadly, how different patient representatives relate to one another becomes an important question (Urbinati and Warren 2008; Lord and Pollak 2010; van de Bovenkamp and Vollaard 2017). We deal with this question by studying the various claims and how the (potential) representatives interact.

This leads us to the following research questions: (1) What actors claim to represent patients, (2) what is their relationship with patients in terms of authorization and accountability, and (3) how does a variety of representatives contribute to the way patients are represented? To answer these questions, we explore the case of Dutch decentralization of care to the municipal level. This is an excellent case to study the multifaceted nature of patient representation. The Netherlands has a long history of involving patient representatives in healthcare decision-making. For example, the Dutch government has encouraged the formal involvement of POs and client councils (councils representing clients on all kinds of issues concerning the healthcare organizations’ policy) in decision-making on both the national level and healthcare provider level (van de Bovenkamp and Trappenburg 2011; Schillemans, van de Bovenkamp, and Trappenburg 2016). Meanwhile, besides POs and client councils, other actors are also claiming to represent patients (van de Bovenkamp and Zuiderent-Jerak 2015; van de Bovenkamp and Vollaard 2017). In January 2015, with the implementation of the Participation Act (concerning social assistance and labour reintegration of people with certain disabilities), the Youth Act (involving social and health policies for those under eighteen, including mental healthcare), and an extended Social Support Act (aiming to help the elderly and people with disabilities to live independently and participate actively in society) saw a major transfer of tasks from the national to the municipal level. Because of the magnitude of this reform, which affected diverse groups of vulnerable patients, a wide variety of representatives were active in the public debate on both the national and local level. All this makes Dutch healthcare decentralization an especially interesting case to study patient representation.

In the next section we describe our methods. Then we report on our results. In the discussion section we reflect on the lessons learned for the debate on patient representation.

## Methods

We used multiple methods to conduct our study. First, to identify the actors claiming to be representatives on the national level, where the decentralization process was heavily debated, we studied relevant documents (e.g., government policy documents and letters sent to parliament by actors claiming to represent patients, including correspondence from professional bodies, patient organizations, and health insurers) by conducting searches of the official government database. In addition, we studied the websites of the actors we identified in this phase as potential representatives to search for additional information on their claims.

Second, we selected two municipalities to study representation on the local level: one medium-sized, economically vibrant university city in the heavily populated conurbation in the west of the country and one small amalgamated municipality in the rural, economically declining north. We selected these municipalities because they have to deal with differing social issues which would give us insight into the multifaceted nature of representation. Our data collection methods included document analysis and interviews. The documents were selected by searching the municipal databases and included decentralization proposals by local executive boards. In addition, we examined the formal contributions of actors trying to influence policymaking (e.g., reports on consultation rounds and council meeting minutes), local newspapers reporting on decentralization news, and the documents and websites of representatives. We conducted qualitative interviews with actors involved in the decentralization debate in the two cases (n=34). Respondents included aldermen, civil servants, city council members, representatives of patient/elderly/informal care organizations, members of municipal advisory bodies, a local journalist, healthcare professionals, and a director of an intermunicipal organization providing sheltered work. We asked our respondents whom they claimed to represent, what their claim was based on, what kind of authorization and accountability mechanisms they had in place, and about any other actors they worked with or identified as representatives. We asked the aldermen and civil servants about other possible representatives and their contact with them. To put our findings in a broader perspective, we also scanned the literature to identify studies concerning representation in the Netherlands in relation to the decentralization.

Our broad approach to patient representation included actors that claimed to represent patients both explicitly and implicitly, for example, by asking for specific attention for certain groups in the debate. The main codes used in our analysis were: actors making representative claims, the groups they claimed to represent, the basis of the claims, authorization and accountability mechanisms, and the interactions between claim-makers.

## Results

Our study made apparent that many and varied actors claim to represent patients in the decentralization debate. Moreover, some actors also pointed to other actors as possible representatives in future. To gain a better understanding of the multifaceted nature of patient representation, we focus on the variety of representatives, the groups they claim to represent, and the authorization and accountability mechanisms they use. We also analyse the way representatives interact.

### The Variety of Representatives

Representatives of patients in the decentralization debate include patient, elderly, and informal carer organizations as well as parliamentarians, municipal council members, municipal advisory councils, and self-appointed societal representatives such as professionals and their organizations, health insurers, and religious leaders. All use a variety of authorization and accountability mechanisms. We discuss these different actors in turn.

#### Patient Organizations

POs claim to represent patients in the decentralization debate. In the Netherlands there are hundreds of national disease-specific POs that provide information, peer support, and representation on disease-specific issues. Often these disease-specific organizations have regional or local branches providing peer support and information to patients. Other POs cater for general patient interests on the regional and local level. Disease-specific POs are organized in a number of national umbrella organizations which are involved in general policy issues, such as decentralization (van de Bovenkamp 2010).

The national umbrella organizations make more-or-less generic representative claims; for instance, one such organization claimed to perform its interest representation activities in the name of “everyone who needs care now or in the future” (NPCF 2014).^1^ Other umbrella organizations make more specific claims for people with a physical handicap, mental disability, or chronic condition (Iederin 2017 ), or healthcare users in the region (Zorbelang Nederland 2017). These national umbrella organizations were active in the national debate on decentralization and called for special attention for the interests of patients or vulnerable people confronted with the effects of decentralization (Wind 2013; Ieder(in) et al. 2014; Ieder(in) et al. 2015).

On the local level, various POs were active. Their claims focused on specific groups, such as the elderly, informal carers, and mental health patients. Our interviews revealed that people active in these organizations on this level are mostly volunteers who are often patients or elderly themselves. However, respondents noted that these organizations struggle to find people willing to take up an active role in their organization. Sometimes professional employees are also active in these organizations. The PO interviews and documents show that active local representatives partly base their claims on experiential knowledge in the case of people who have experience as a service user themselves. In addition, they keep in touch with the groups they claim to represent through, for instance, meetings with members and call centres.

POs use several authorization and accountability mechanisms. Most disease-specific POs are associations that offer membership to patients. Members can direct and hold their organization to account through general meetings. National umbrella organizations offer membership to disease-specific organizations, and they can also use membership as a mechanism for authorization and accountability (Oudenampsen et al. 2008; van de Bovenkamp, Trappenburg, and Grit 2010; Fischer and van de Bovenkamp forthcoming). In addition, national umbrella organizations maintain direct contact with patients through patient panels, call centres, and websites where patients, their family, or carers can post experiences (Patientenfederatie 2017; Iederin 2017; Zorgbelang Nederland 2017). Our interviews showed that local and regional patient organizations also draw on direct contact with patients by organizing meetings, panels, contact points, and polls. These contacts can be used as input to authorize the claims POs make and offers patients the chance to hold POs to account. In addition, at least part of the representation efforts of POs are made public on their website and in the media, which again offers patients the opportunity to hold POs to account.

For instance, if you have to say something in the press about something or other, we get phone calls right away, like, “He speaks for the elderly, but I don’t agree with what he said.” (respondent, patient organization).

In general, however, according to our respondents, patients show limited interest in holding POs to account. Moreover, our interviews showed that much of the POs’ representation efforts take place informally through contact with policymakers and aldermen. It is difficult to hold such informal representation work to account as it happens outside the public eye.

#### Elected Representatives

Besides the POs, elected political representatives, parliamentarians, and local council members also claimed to represent patients or—more broadly defined—service users in the debate. Our analysis showed that these elected representatives sometimes make generic claims about “the people” and at other times focus on specific groups affected by the decentralization.

The claims of elected representatives are partly based on their institutional position. However, respondents note that some council members base their claims on their knowledge of the problems and preferences of service users that they have gained through direct contact with citizens, site visits to care organizations, contacts with other representatives, or their own experience as a care professional or service user.

For parliamentarians and city councillors, elections are an important mechanism for authorization and accountability. In the Netherlands, national elections generally take place every four years (or earlier in the case of problems in a coalition). Healthcare is one of the most important issues for voters both nationally and locally (TNS-NIPO 2017; SCP 2017). A couple of problems can be identified with this authorization and accountability avenue, however—an important one being low voter turnout. Voter turnout has declined steadily in recent decades, although there was a high turnout in the last national election (81.9 per cent compared to 74.6 per cent four years earlier). On the local level, voter turnout is considerably lower: 54.0 per cent at the last local elections (Kiesraad 2017). It is important to note that certain groups, especially the less educated, vote less often than other groups in elections (Bovens and Wille 2011; TNS-NIPO 2017). The danger arising from this is that some voters are catered for less than others. In the case of decentralization this is especially important because these groups display differences in terms of usage and preferences for social and health services (SCP 2017).

Our empirical work shows that elected representatives also use other mechanisms for authorization and accountability to differing degrees. Concerning the former, local councillors, for instance, use direct day-to-day contacts with citizens, working visits to healthcare providers, and organizing contact points or discussions with POs or professionals. Some draw on their own experience as service user or professional:

Time and again we come across the arrogance of institutions; it’s really unbelievable. Then it’s convenient that you’ve been around as a service user for a couple of years. (city council member)

In between and besides elections, the accountability of the work of parliamentarians and city council members is ensured in several ways. Parliamentary and council meetings are open to the public, minutes of meetings are available, and discussions concerning decentralization are reported by the media. However, our analysis showed that newspapers reported on decentralization only to a limited extent on the local level. Also, in this case respondents report that citizens show only sporadic interest in accountability in practice. In general, citizens do not seem to be very interested in accountability:

People come to me with lots of questions for help (…) but accountability? No, I’ve never had the feeling that people ask for that. People lose interest before they come and ask about accountability. (city councillor)

#### Advisory Councils

On the municipal level there are advisory councils, appointed by the local executive board of the mayor and aldermen. Their job is to give the executive board advice on the Social Support Act, social assistance, and sheltered work. The councils claim to represent various groups affected by decentralization, such as people with disabilities, mental health problems, people on benefits, the elderly, and homeless people. The people active in these councils can be individual service users, representatives of local societal (mostly patient and elderly) organizations, and people with professional expertise (Panhuijzen and Lucassen 2017). As with the claims of elected representatives, the claims of advisory councils are partly based on their institutional position; they are appointed by the executive board to fulfil this function. Our interviews showed that advisory council members also base their claims on shared experience (when members are service users themselves), contact with service users, and professional expertise.

Our interviews also showed that advisory councils partly authorize themselves through experiential knowledge. However, respondents note that similar to POs, it not always easy to find service users willing to participate. The fact that these councils are expected to respond to complicated policy documents and offer advice on those policies adds to this problem as few service users are able to play this role. Therefore, some councils choose to involve people with expertise in policy issues and political decision-making. The downside put forward by respondents is that this strategy can lead to council members lacking everyday practical knowledge. Efforts are made to limit this problem by meeting the members of the organization they belong to (e.g., council members from organizations for the elderly) or establishing contact with service users through volunteer work or site visits. A council member who tried hard to get to know her constituency acknowledged that there are still limits to what she knows.

As a patient I have lots of contact with fellow patients. I have lots of contact with homeless people because I do volunteer work. But I’m not under the illusion that you make it that way, that you really know it all. (former SSA council member).

Advisory councils can be held to account in several ways. Minutes of meetings and reports of their activities and recommendations can in most cases be found on the websites. Again, respondents do not feel there is much interest in accountability. Moreover, similarly to POs, part of their work happens informally and is therefore difficult to give an account of.

A lot happens before the requested recommendation is drawn up. That’s not visible to anybody else [but] you already have a lot of influence. (ex-chairman social support council)

#### Self-Appointed Societal Representatives

To conclude, we identified a number of self-appointed societal representatives in the decentralization debate. Patient representation is not the core task of these representatives and they cannot draw on a formal institutional position to legitimize their claim. However, they do claim to represent the interests of patients at several points in the decentralization debate or are identified by other actors as possible representatives for the future. For instance, on the national level, professional organizations expressed their concerns, especially regarding the decentralization of youth care, to the Ministry of Health, to parliament, and in the public debate (KNMG 2013; NHG/LHV 2013; KNMG 2014). They pointed at the risks for accessibility of care and inequality for “this very vulnerable and dependent group in our society” *(*KNMG 2014, 2). Individual professionals also spoke up, such as the child psychiatrist quoted at the beginning of the paper. The basis of his involvement was his professional identity: “I am a doctor and therefore I am concerned” (Müller 2013). Healthcare insurers are also active in the national debate. They claimed, based on their “great societal responsibility,” to speak up, for instance, in a letter to parliament:

The changes concerning care for the vulnerable elderly, children with serious conditions or people with long-term psychiatric problems demand great precision. (Rouvoet 2014)

On the local level, professionals, such as GPs, were less active at the time of our empirical work. However, respondents acknowledged that GPs and similar professionals can be seen as important representatives in future debates on decentralized services. They are attributed this position based on their close knowledge of the problems patients encounter: “They go to someone’s home and see the misery” (respondent patient organization). Churches were another actor identified as a possible representative of groups confronted by decentralization. Similar to healthcare professionals, they are said to be one of the few actors who have close contacts with groups of people who may not be inclined to speak up for themselves.

It [the church] is one of the few organizations that gets into all kinds of peoples’ homes […] I mean in most cases a church arranges its house visits properly. They go to peoples’ homes and see what happens there, talk to people. So it is one of the organizations that can be the first to spot [problems]. (chairman local advisory council for the Social Support Act)

Also similar to professionals, churches are generally not very active on the local level yet, although this differs between municipalities (Noordegraaf 2012).

Being self-appointed representatives, these actors do not have formal authorization and accountability mechanisms in place. However, they can draw on informal contacts with their “constituency.” As said, the respondents consider professionals and churches to have important knowledge which allows them to represent patients. They can draw on their day-to-day interaction with groups affected by decentralization and can therefore spot problems that users are struggling with. Furthermore, in some cases alternative means of authorization are organized. For instance, some psychiatrists started a petition against the decentralization of youth care as they felt that it would negatively impact mental healthcare for children and adolescents with a psychiatric condition. Their call for “help to ensure good care for children” was signed by, among others, parents, psychologists, boards of directors of mental-health institutions, and teachers. This petition can be seen as an authorization of the representative claim (original website http://www.petitiejeugdggz.nl/, accessed February 14, 2017).^2^ The claims of these individual professionals and the professional organizations trying to influence policies were also reported in the media and on websites, and official letters sent to the ministry were publicly available. Therefore, these claims were made accountable. On the local level, professionals and others with everyday experience were active only to a limited extent, but our document analysis showed that they did deliver input during consultation rounds and in the media, which ensured the accountability of their claims.

#### Combining Patient Representation Claims

As the above descriptions have made clear, many and various actors are claiming to represent patients. Some make general representative claims (e.g., the people and service users in general), others’ claims are more specific and focus on vulnerable groups who often remain silent themselves (e.g., youth with mental health problems and the frail elderly). Seen this way, the different claims add to each other and can help ensure that a large range of voices are heard.

Our document analysis and interviews show that in some cases representatives work together to strengthen their claims. They do so by sharing information and directing their claims to decision-makers together. For example, on the national level, patient and informal carer organizations have combined their efforts to influence the decentralization policy on several occasions by sending joint letters to parliament (Ieder(in) et al. 2014; Ieder(in) et al. 2015). Also, on the local level, representatives seek contacts to strengthen a claim:

It has a bigger impact when the local council says it. It [sharing information] is convenient for municipal council members, because all of a sudden they have more ammunition to shoot with. I think you can have more influence that way. (employee client organization)

On the local level, respondents noted that working together happens only sporadically. They explained that organizational issues stand in the way of cooperation because many actors are not organized at the local level, the level where decisions are now taken. Many of them, including patient and professional organizations, are organized nationally and regionally. This makes it difficult to direct their representation efforts to the local level, let alone ensure collaboration with other local representatives.

Another reason why local representatives work together infrequently is because most direct their efforts to the executive board of the municipality. This limits information sharing between representatives as this contact happens outside the public debate. For instance, our document analysis and interviews showed that representatives such as POs seldom use their right to speak at council meetings. In turn, local councils seldom use the right to organize public hearings to which they can invite other claim-makers on the subject of decentralization.

The above results in fragmented local representation in practice. On the one hand, this means that concerns can be expressed repeatedly and attention can be focused on specific groups. On the other hand, the danger is that fragmentation can lead to instrumental use, as different claims can be used to silence each other. For example, the alderman may involve some advisory councils at an early stage of policy development, earlier than the elected local council. Respondents note that this can put elected representatives in a difficult position because proposals have already been discussed with other representative claim-makers before they have received the proposals.

## Discussion

With this overview of representatives, we have identified several important points that can contribute to the discussion on patient representation.

When studying the subject of patient representation, it is important to take a broad perspective, moving beyond the “usual suspects”—the POs—to bring into view the large variety of representatives. This variety has the important advantage of making it possible for the voices of patients to be heard through multiple channels in the public debate. Moreover, such variety can help do justice to the wide variations in patient preferences. Often patient representatives are asked to contribute “the patient perspective” in decision-making (van de Bovenkamp and Zuiderent-Jerak 2015). Moreover, the POs try to take a clear position in policy debates in order to strengthen their influence (Fischer and van de Bovenkamp forthcoming). However, research has demonstrated that there is no singular “patient perspective.” Patients find different things important in their care, and this has an impact on their policy preferences (Callon and Rabeharisoa 2004; Barbot 2006; Epstein 2008; van de Bovenkamp and Dwarswaard 2017). Therefore, no one voice can claim to represent all patients. The fact that various claim-makers make different contributions to the debate can help democratize the process and lead to more legitimate outcomes (Parkinson 2004). The variety of patient representatives can be viewed positively in this regard, as representatives can speak for different patient groups. For instance, it has been identified that only active professionalized patients are capable of becoming active representatives in POs in the formal decision-making processes. This may limit their ability to speak for less active patient groups who remain silent (Baggott and Forster 2008; van de Bovenkamp 2010; van de Bovenkamp and Zuiderent-Jerak 2015). Healthcare professionals are among the actors identified by our respondents who can limit this problem because they are among the few to come into contact with certain groups of vulnerable patients. Of course this claim can also be viewed critically; professionals may have interests at heart other than the interests of their patients. This makes it important to have a debate on the acceptance of representative claims. Although multiple claims to representation carry the risk of instrumental use, as decision-makers can pick and choose strategically the representative claim closest to their own preferences, multiple claims may also strengthen democratic decision-making when made in the public debate. In this case, competing claims need explication before acceptance, a step that can strengthen the representative relationship. For this to happen it is important to debate these claims in public. That much representative work happens informally—as we found in our case study—can be considered problematic in this regard.

Of course, the actors actually capable of representing patients depends on the specific national context and the policy issue at stake. Regarding the latter, in the case of Dutch decentralization, representatives expressed their concern for basic issues such as access to care. Also, other actors besides POs can identify and address problems. This might be different in the case of concrete quality improvement projects in a healthcare organization or when setting the research agenda as then it is more important to tap into the experience of patients themselves (Pols 2013; Vennik et al. 2016). However, in these cases as well, it is important to do justice to the variety of patient preferences. This warrants reflection on how to tap into the variety of preferences and the role POs can play in that regard. POs could, for example, act as intermediaries bringing decision-makers in touch with their members (van de Bovenkamp and Vollaard 2017).

The paper also shows that the democratic quality of patient representation can be enhanced by discussion and reflection on the work of POs and other representatives. Issues that should be included in such discussion include how claim-makers relate to one another and if and how they want to work together. There are some examples where working together happens in practice, but this study showed that improving such practices might require reorganization and making choices regarding representation efforts (e.g., who to direct their claim to and whether to do so informally or in public). In addition, it is crucial to think about how to ensure the relationship with those whom the actors claim to represent (i.e., patients) in terms of authorization and accountability. Especially the latter can be considered problematic in practice. Although representatives feel the need to account for their actions, those whom they claim to represent hold them to account only to a limited extent. In light of this limited interest in the accountability of representatives, an important subject for further research would be to investigate the patients’ acceptance of the different claims.

## Footnotes

1 Since the debate on decentralization began, this organization has changed its name and mission statement to tone down their representative claim a little to “everyone who needs care” ( Patientenfederatie 2017).

2 The petition was eventually signed by 96,209 people (originally found at http://www.petitiejeugdggz.nl/, accessed February 14, 2017).
